# How histopathological diagnosis interacts with kidney ultrasound parameters and glomerular filtration rate

**DOI:** 10.1007/s11739-024-03711-7

**Published:** 2024-09-13

**Authors:** Simeone Andrulli, Antonietta Gigante, Michele Rossini, Pierluigi D’Angio’, Gisella Vischini, Franca Luchetta, Filippo Aucella, Giovanni Valsecchi, Barbara Infante, Maria Giovanna Vario, Domenico Giannese, Antonio Granata, Elisabetta Moggia, Guido Gembillo, Rosario Cianci, Mario Bonomini, Flavia Manenti, Roberta Lazzarin, Brigida Di Renzo, Fulvia Zanchelli, Maurizio Garozzo, Massimo Manes, Yuri Battaglia, Raffaela Sciri, Marco De Fabritiis, Marco Quaglia, Gioacchino Li Cavoli, Enrica Gintoli, Maria Maddalena Conte, Maurizio Borzumati, Luisa Benozzi, Giovanna Pasquariello, Giovanni Andrulli, Marco Leoni, Giuseppe Seminara, Valentina Corbani, Gianmarco Sabiu, Arcangelo Di Maggio, Rosa Maria Pollastro, Loreto Gesualdo, ITA-KID-BIOPSY Group

**Affiliations:** 1 Associazione Italiana Ricercare per Curare ODV ETS (AIRpC), Lecco, Italy; 2 Department of Translational and Precision Medicine, Sapienza University of Rome, Rome, Italy; 3 Nephrology, Dialysis and Transplantation Unit, Department of Emergency and Organ Transplantation, University of Bari Aldo Moro, Bari, Italy; 4 Maria Santissima Addolorata Hospital, Nephrology and Dialysis Unit, Eboli, Italy; 5 Nephrology Dialysis and Renal Transplant Unit, IRRCS Azienda Ospedaliero-Universitaria, Bologna, Italy; 6 Belcolle Hospital, Nephrology and Dialysis Unit, Viterbo, Italy; 7 Nephrology and Dialysis Unit, “Casa Sollievo Della Sofferenza” Foundation, Scientific Institut for Reserch and Health Care, San Giovanni Rotondo, Italy; 8 Nephrology, Dialysis and Transplantation Unit, Department of Biomedical Sciences, Foggia, Italy; 9 Nephrology and Dialysis Unit, Villa Sofia Cervello United Hospitals, Palermo, Italy; 10 Nephrology, Dialysis, Transplantation, Azienda Ospedaliero Universitaria Pisana, Pisa, Italy; 11 Nephrology and Dialysis Unit, San Giovanni di Dio, Agrigento, Italy; 12 Nephrology and Dialysis Unit, Ospedale S Croce, Cuneo, Italy; 13 Nephrology and Dialysis Unit, Department of Clinical and Experimental Medicine, University of Messina, Messina, Italy; 14 Nephrology and Dialysis Unit, Department of Medicine, G. d’Annunzio University, SS. Annunziata Hospital, Chieti, Italy; 15 Nephrology and Dialysis Unit, San Salvatore Hospital, Pesaro, Italy; 16 Nephrology and Dialysis Unit, Ospedale San Giacomo Apostolo, Castelfranco Veneto, Italy; 17 Nephrology and Dialysis Unit, Ospedale A. Perrino, Brindisi, Italy; 18 Nephrology and Dialysis Unit, Ospedale Santa Maria delle Croci, Ravenna, Italy; 19 Nephrology and Dialysis Unit, Santa Marta and Santa Venera Hospital District, Acireale, Italy; 20 Nephrology and Dialysis Unit, Umberto Parini Hospital, Aosta, Italy; 21 Nephrology and Dialysis Unit, Ospedale S. Anna, Ferrara, Italy; 22 Nephrology and Dyalisis Unit, S. Maria della Misericordia Hospital, Perugia, Italy; 23 Nephrology and Dialysis Unit, Morgagni-Pierantoni Hospital, Forlì, Italy; 24 SCDU Nefrologia e Dialisi, AOU “SS Antonio e Biagio e Cesare Arrigo”, Università del Piemonte Orientale (UPO), Alessandria, Italy; 25 Department of Nephrology Dialysis Renal Transplantation, Civic Hospital, Palermo, Italy; 26 Nephrology and Dialysis Unit, Arcispedale Santa Maria Nuova di Reggio Emilia, Reggio Emilia, Italy; 27 Nephrology and Dialysis Unit, University Hospital Maggiore della Carità, Novara, Italy; 28 Nephrology and Dialysis Unit, Castelli Hospital ASL VCO, Verbania, Italy; 29 Nephrology and Dialysis Unit, SS. Trinità Hospital, Borgomanero, Italy; 30 Nephrology and Dialysis Unit, Osp. S. Chiara, Pisa, Italy; 31 Bocconi University, Milan, Italy; 32 Nephrology and Dialysis Unit, Ospedale Regina Apostolorum, Albano Laziale, Italy; 33 Nephrology and Dialysis Unit, Cannizzaro Hospital, Catania, Italy; 34 Nephrology and Dialysis Unit, Sant’Andrea Hospital, La Spezia, Italy; 35 Nephrology and Dialysis Unit, ASST Fatebenefratelli-Sacco, Milano, Italy; 36 Nephrology and Dialysis Unit, SS. Annunziata, Taranto, Italy; 37 Department of Translational Medical Sciences, University of Campania Luigi Vanvitelli, Naples, Italy

**Keywords:** Kidney failure, Chronic, Histology, Proteinuria, Diabetes mellitus, Ultrasonography

## Abstract

The evaluation of estimated GFR (eGFR) is a pivotal staging step in patients with chronic kidney disease (CKD), and renal ultrasound plays an important role in diagnosis, prognosis and progression of CKD. The interaction between histopathological diagnosis and ultrasound parameters in eGFR determination has not been fully investigated yet. The study examined the results of native kidney biopsies performed in 48 Italian centers between 2012 and 2020. The primary goal was if and how the histopathological diagnosis influences the relationship between ultrasound parameters and eGFR. After exclusion of children, patients with acute kidney injury and patients without measure of kidney length or parenchymal thickness, 2795 patients have been selected for analysis. The median values were 52 years for patient age, 11 cm for bipolar kidney diameter, 16 mm for parenchymal thickness, 2.5 g/day for proteinuria and 70 ml/min/1.73 m^2^ for eGFR. The bipolar kidney diameter and the parenchymal thickness were directly related with eGFR values (R square 0.064). Diabetes and proteinuria were associated with a consistent reduction of eGFR, improving the adjusted R square up to 0.100. Addition of histopathological diagnosis in the model increased the adjusted R square to 0.216. There is a significant interaction between histopathological diagnosis and longitudinal kidney diameter (*P* 0.006). Renal bipolar length and parenchymal thickness are directly related with eGFR. The magnitude of proteinuria and histopathological kidney diagnosis are associated with eGFR. The relationship between kidney length and the level of eGFR depends on the nature of the kidney disease.

## Introduction

Renal Ultrasound (US) plays an important role in the diagnosis and progression of chronic kidney disease (CKD) [1]. The evaluation of CKD is classified based on the glomerular filtration rate (GFR) estimate, urinary abnormalities, and ultrasound structural kidney abnormalities. When CKD is suspected or diagnosed, longitudinal kidney diameter, parenchymal thickness and echogenicity grading are the first measures to be gathered, through renal US, as first imaging tool [2]. CKD can be associated with different values of longitudinal kidney diameters. It increases in polycystic kidney disease, in myeloma cast nephropathy, in amyloidosis, and in the beginning of the diabetic Kimmestiel-Wilson nephropathy. Contrarily, it decreases in many other nephropathies, such as chronic glomerulonephritis, nephroangiosclerosis and chronic ischemic nephropathy.

The estimated GFR (eGFR) in place of its measure [3, 4] is a pivotal step in the CKD staging and can be performed by using various approaches, like the Cockroft-Gault [5] and the Modification of Diet in Renal Disease (MDRD) equations [6]. In the clinical practice context, the Cockroft-Gault equation has been progressively abandoned, partially because it requires the knowledge of the patient weight, often unavailable at the laboratory level and, in addition, it overestimates the true GFR at high values of body mass index [7]. On the other side, the first MDRD eGFR equation [8] has been improved to take into account three subsequent needs: a standardized measurement of creatinine [9], a simplified equation (four variables in place of the first six) [9], and a higher accuracy at GFR values higher than 60 ml/min/1.73 m^2^ [6]. In 2012 [10], 2014 [4] and 2021 [11], other equations developed in different populations were published, and the Chronic Kidney Disease Epidemiology Collaboration (CKD-EPI) equations have been used more frequently for general clinical purpose. The 2021 CKD-EPI equation offered the opportunity to take out the race information from the eGFR estimation [11]. Unfortunately, this race-free equation may result in substantial change in eGFR estimation, in CKD reclassification, in kidney and cardiovascular prognosis [12–14], and in substantial error in comparison with the measured GFR, also among kidney transplant recipients [15].

Future research should focus on the lack of a more precise eGFR equations at the individual level [16], and the risk of a misleading indexing of glomerular filtration rate for body surface area in obese patients [17].

The aim of this study, instead, is to define the relationship between the kidney diameters measured in vivo with ultrasound and the estimated GFR according to the CKD-EPI 2009 equation [6], taking into account the role of the histopathological diagnosis available with native kidney biopsy.

## Materials and methods

### Patient selection

The invited Italian study centers and the patients enrolled in this study are described in detail in our previous work [18]. Briefly, as this was a cross-sectional observational multi-center study, the enrollment criteria were not questioned. Consequently, all of the consecutive patients undergoing a native kidney biopsy during the active recruitment period were considered eligible and there were no a priori exclusion criteria. In relation with the aim of this study, secondary exclusion criteria were pediatric patients (age at kidney biopsy less than 18 years), unstable patients for acute kidney injury (AKI) or AKI in patients on chronic kidney disease (CKD), unavailability of eGFR or its estimated value higher than 200 ml/min, unavailability of kidney length or of parenchymal thickness of biopsied kidney.

Data collection was centralized and made use of an ad hoc web-based database linked to the Italian Renal Biopsy Registry (http://www.irrb.net/).

All of the patients gave their written informed consent; the study protocol was approved by the Ethics Committee of Bari University and implemented in accordance with the principles of the Declaration of Helsinki. It was not appropriate to involve patients or the public in the design, or conduct, or reporting, or dissemination plans of our research. This independent study without any sponsorship was registered with ClinicalTrials.gov (No. NCT04948593).

### Outcomes

The primary goal of this study was to define the relation between the renal length and parenchymal thickness measured with ultrasounds and the eGFR, taking into account the role of the histopathological diagnosis made by native kidney biopsy.

### Variables

Relevant patient-related covariates and factors recorded included age, gender, diabetes, the clinical presentation of their renal disease, the presence of renal failure, the eGFR according to CKD-EPI equations [9], the bipolar longitudinal diameter and the parenchymal thickness of the biopsied kidney, the magnitude of proteinuria and the histopathologic kidney diagnosis. The histopathologic kidney diagnosis, defined according to our previous work [18], was treated in the statistical analysis as a categorical variable.

Ultrasound parameters were measured in the biopsied kidney, thus more frequently on the left side (95% of patients), on the midaxillary line with the patient in lateral decubitus. Parenchymal thickness was measured and reported where it was minimum in value, avoiding Bertin's columns.

To avoid bias, proteinuria, eGFR and ultrasound parameters were measured before the native kidney biopsy.

The diabetic variables were considered at three levels. The clinical diabetes status (yes/no) was defined according to the clinical diagnosis of diabetes without the knowledge of the histopathologic kidney diagnosis. In addition, the type [19] and the severity [20] of diabetic nephropathy according to Mazzucco G et al. [19] and to Tervaert TW et al. [20], respectively, were also considered.

### Statistical analysis

For descriptive purposes, quantitative variables were analysed using their median values and the 10th and 90th percentiles, as indexes of central tendency and variability, respectively. Categorical variables were analysed as absolute numbers and percentages.

For inferential purposes, multivariate analysis of variance was performed, using the eGFR as dependent variable, according to CKD-EPI equation [9] expressed in ml/min/1.73 m^2^. To investigate the role of the various covariates, a step-by-step approach was used starting from the ultrasound parameters, such as the kidney bipolar diameter and parenchymal thickness. According to the suggestion of Lucisano et al. [21], we have considered also the role of the kidney length indexed for body height compared with the kidney length alone. The next step was adding two covariates easily available before kidney biopsy, such as the clinical diabetes status (yes/no) and proteinuria (g/day) values. To investigate the slope between US parameters and eGFR in patients with clinical diabetes compared with patients without clinical diabetes, we tested the interaction term clinical diabetes status by kidney length. Thus, we tested the histopathologic kidney diagnosis, as categorical variable, and its interaction with the US biopsied kidney length. Finally, we added in the model the type [19] and severity [20] of diabetic nephropathy. The amount of explained variance, through the adjusted R square, was used as goodness of fit. The partial Eta square of each covariate was used to test the relative net impact of each one compared to the others.

All the analyses were performed using the Statistical Package for Social Sciences (SPSS for Windows, version 23.0).

## Results

**Fig. 1:**
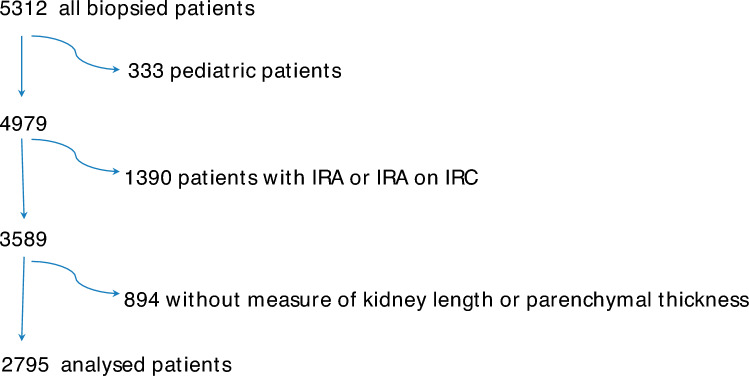
Selection of study sample. The final analysed sample of 2795 patients was selected from a pool of 5312 biopsied patients, after exclusion of children, patients with acute kidney injury (AKI) or AKI on chronic kidney disease (CKD) and patients without measure of kidney length or parenchymal thickness

This study involved 48 Italian centers (see Appendix) and 5312 patients, enrolled from the 3rd of January 2012 to the 4th of August 2020. After exclusion of children (333 patients, 6%), the patients with acute kidney injury (AKI) or AKI on CKD (1390 patients, 26%) and patients without measure of kidney length or parenchymal thickness (894 patients, 17%), the final sample of 2795 patients (53%) was selected for analysis from the pool of 5312 biopsied patients (Fig. 1). Thus 2795 patients, with one kidney biopsy for each patient, constituted the study group for this report. The main characteristics of the analysed patients were shown in Table 1. The median values were 52 years for patient age, 11 cm for bipolar kidney diameter, 16 mm for parenchymal thickness, 2.5 g/day for proteinuria and 70 ml/min/1.73 m^2^ for eGFR. Male were prevalent (60.5%), with a clinical diagnosis of diabetes status in 14.9% of the cases. Urinary abnormalities (49.2%) and nephrotic syndrome (39.4%) were the more common clinical presentations of studied patients.

**Table 1:** Patient characteristics of the 2795 studied patients

*Quantitative variables*:					
	Percentiles				
	10th	25th	50th	75th	90th
Age (years)	28	40	52	64	72
Body Weight (Kg)	56	65	74	84	96
Body Mass Index (BMI) (kg/ m^2^)	21	23	26	29	33
Systolic blood pressure (mmHg)	110	120	130	140	150
Diastolic blood pressure (mmHg)	62	70	80	80	90
Bipolar kidney diameter (cm)	9.8	10.2	11.0	11.7	12.2
Parenchymal thickness (mm)	10	13	16	19	20
Creatinine (mg/dL)	0.7	0.8	1.1	1.7	3.0
eGFR (ml/min/1.73 m^2^)	21	40	70	98	115
Proteinuria (g/day)	0.5	1.0	2.5	5.0	9.0

*Categorical variables:*	%
Gender (M/F)	60.5/39.5
Age ≥ 65 years	23.8
Diabetes (Yes)	14.9
Hypertension (Yes)	53.1

A valuable variability was present for age, BMI, ultrasound kidney measures and proteinuria

**Table 2:** Histopathological diagnoses of 2795 native kidney biopsies. The three more frequent diagnoses were IgA Nephropathy (IgAN), Membranous Nephropathy (MN) and Focal and Segmental GlomeruloSclerosis (FSGS) Table 2

	*n*	%
IgA nephropathy	491	17.6
Membranous nephropathy	432	15.5
Focal segmental glomerulosclerosis	293	10.5
Not defined	292	10.4
Hypertension and hischemic renal injury	208	7.4
Diabetic nephropathy	208	7.4
Minimal change disease	202	7.2
Lupus nephritis	181	6.5
Amyloidosis	104	3.7
Tubulointerstitial disease	80	2.9
Normal kidney	56	2.0
ANCA-associated vasculitis	54	1.9
Membranoproliferative glomerulonephritis	37	1.3
Light chain deposition disease	29	1.0
Hereditary glomerulopathies	28	1.0
C3 nephropathy	19	0.7
Henoch Schoenlein purpura	18	0.6
Myeloma cast nephropathy	13	0.5
Immunotactoid fibrillary nephropathy	10	0.4
Thrombotic microangiopathies	8	0.3
Cryoglobulinemic glomerulonephritis	8	0.3
Acute post infectious glomerulonephritis	7	0.3
Inadequate material	5	0.2
Storage disease	4	0.1
Goodpasture	3	0.1
Other	5	0.2
Total	2795	100.0

### Multivariate analysis

The Table 3, Panel A shows how the ultrasound kidney parameters, such as the bipolar kidney diameter and the parenchymal thickness, are associated with eGFR values. As expected, the B coefficients of both ultrasound parameters were positive, indicating a direct association with the eGFR values. From a clinical perspective, for each incremental centimeter in renal length, we can expect an increase of 6.89 ml/min/1.73 m^2^ of eGFR. The adjusted model R square value is 0.064, showing that the ultrasound parameters, together with the gender variable, explain only 6.4% of the eGFR variability. This percentage is not improved by using kidney length and parenchymal thickness *indexed for body height* in place of the kidney length alone (data not shown).

**Table 3:** Multivariate analysis of variance of the eGFR based on ultrasound kidney parameters, such as the bipolar kidney diameter and the parenchymal thickness (Panel A) and, in addition, on clinical diabetes status and proteinuria values (Panel B)

Variable	B	SE	*t*	*P* value	95% CI		Eta square
					Lower	Upper	
*Panel A:*							
Bipolar kidney diameter (cm)	6.89	0.67	10.34	< 0.001	5.58	8.19	0.037
Parenchymal thickness (mm)	0.70	0.17	4.20	< 0.001	0.37	1.03	0.006
Gender (Male)	− 7.91	1.32	− 5.99	< 0.001	− 10.50	− 5.32	0.013
Model adjusted R square of 0.064							

Variable	B	SE	*t*	*P* value	95% CI		Eta square
					Lower	Upper	
*Panel B:*							
Bipolar kidney diameter (cm)	7.80	0.66	11.81	< 0.001	6.50	9.09	0.048
Parenchymal thickness (mm)	0.69	0.16	4.22	< 0.001	0.37	1.01	0.006
Gender (Male)	− 6.92	1.30	5.33	< 0.001	− 9.47	− 4.38	0.010
Clinical Diabetes (Yes)	− 16.86	1.78	− 9.48	< 0.001	− 20.35	− 13.37	0.031
Proteinuria (g/day)	− 0.66	0.15	− 4.42	< 0.001	− 0.95	− 0.37	0.007

Model adjusted R square of 0.100

In the Panel B, the adjusted R square value of the model increased from 0.064 to 0.100, with the persistent major contribution of the bipolar kidney diameter (Eta square of 0.048). Male gender, clinical diabetes status and proteinuria were associated with a significant and a consistent reduction of eGFR value (see B coefficients)

Adding other two covariates easily available before and without kidney biopsy, such as the clinical diabetes status and proteinuria values (Table 3, Panel B), the adjusted model R square value increased from 0.064 to 0.100, with the persistent major contribution of the bipolar kidney diameter (Eta square of 0.048). Interestingly, male gender, diabetes status and proteinuria were associated with a significant and a consistent reduction of eGFR value (see B coefficients in Panel B of Table 3). The interaction of clinical diabetes with ultrasound kidney parameters (bipolar length and parenchymal thickness) was not statistically significant (*P* value 0.389 and 0.819, respectively), meaning that the slope between the ultrasound kidney parameters and eGFR is not different between patients with and without clinical diabetes. Thus, for each incremental centimeter in renal length, we can expect the same increase of eGFR in both patients with and without clinical diabetes.

**Fig. 2:**
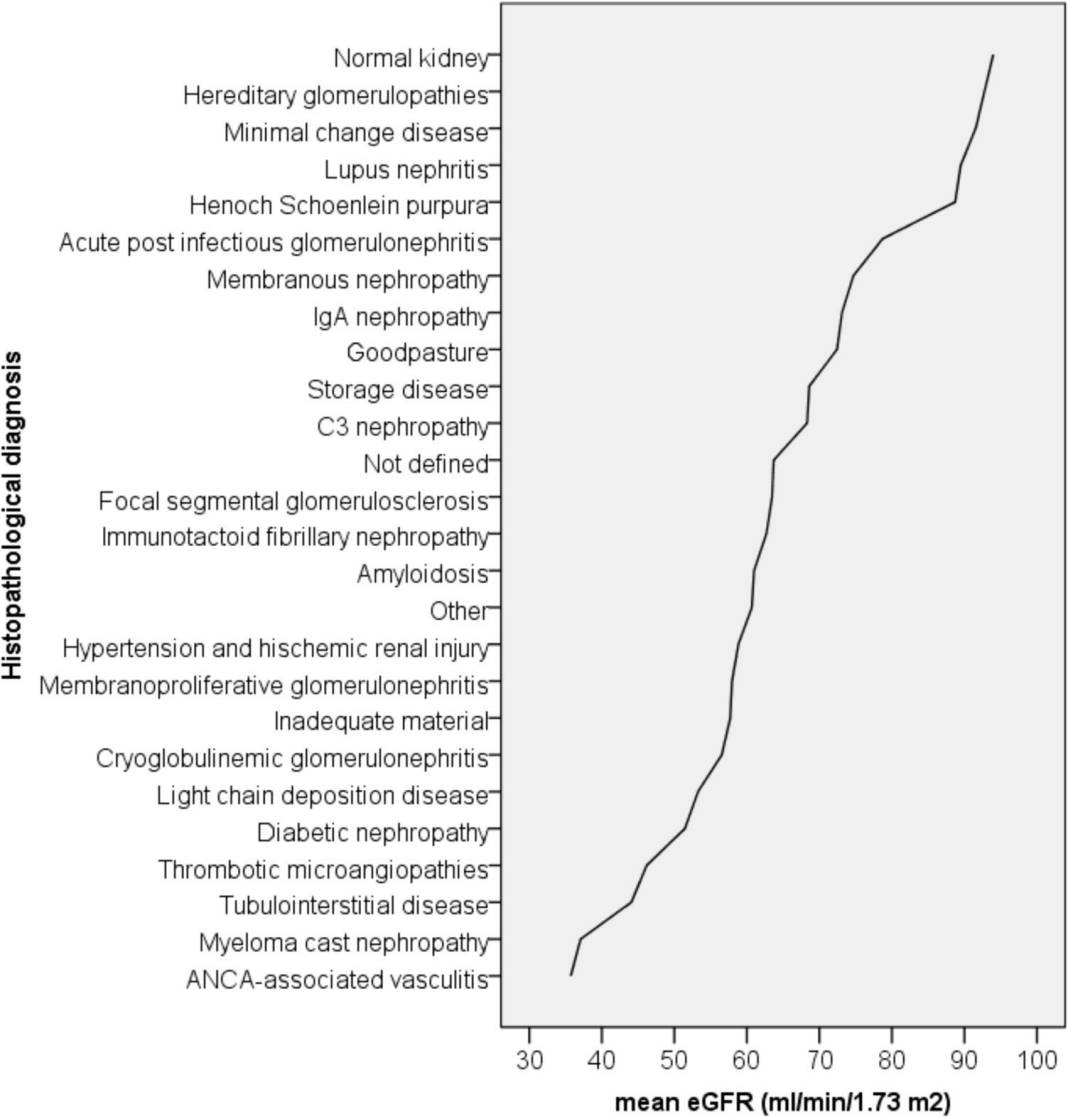
Distribution of mean eGFR values by the histopathological diagnosis, at the time of kidney biopsy. Some histopathological diagnoses were associated with a near normal eGFR value, as in cases of normal kidney, minimal chance disease (MCD) or hereditary glomerulopathies. On the other side, other diseases, as small vessel vasculitis and myeloma cast nephropathy were more frequently associated with low eGFR values

The histopathological diagnosis added a lot of information on the eGFR/ultrasound parameters relationship (Table 4, Panel A). Indeed, the adjusted model R square value increased consistently from 0.100 to 0.216, with the major contribution made now by the histopathological diagnosis (*P* < 0.001, Eta square of 0.022). Moreover, as expected, there was a significant interaction of histopathological diagnosis with longitudinal kidney diameter (*P* = 0.006, Eta square of 0.017) suggesting that the association of kidney length with the level of eGFR was dependent on the nature of kidney disease. The histopathological diagnosis, at the time of kidney biopsy, influenced also the distribution of mean eGFR values (Fig. 2): some histopathological diagnoses were associated with a nearly normal eGFR value, as in case of normal kidney, of minimal chance disease (MCD) or of hereditary glomerulopathies. On the other side, small vessel vasculitis and myeloma cast nephropathy were more frequently associated with low eGFR values.

**Table 4:** Multivariate analysis of variance of the eGFR based on ultrasound kidney parameters, diabetes status, proteinuria value and histopathological diagnosis (Panel A) and in addition on renal pathology score (Panel B)

Variable	SE	B	*t*	*P* value	95% CI		Eta square
					Lower	Upper	
*Panel A:*							
Bipolar kidney diameter (cm)	3.22	9.17	2.85	0.004	2.85	15.49	0.003
Parenchymal thickness (mm)	0.16	0.64	4.12	< 0.001	0.34	0.94	0.006
Gender (Male)	1.27	− 3.72	− 2.93	0.003	− 6.21	− 1.23	0.003
Proteinuria (g/day)	0.15	− 1.15	− 7.53	< 0.001	− 1.45	− 0.85	0.020
Clinical Diabetes (Yes)	2.18	− 8.17	− 3.75	< 0.001	− 12.45	− 3.90	0.005
Histopathological diagnosis				< 0.001			0.022
Histopathological diagnosis * bipolar kidney diameter interaction				0.006			0.017
Model adjusted R square of 0.216. Histopathological diagnosis is a categorical variable							

Variable	B	SE	*t*	*P* value	95% CI		Eta square
					Lower	Upper	
*Panel B:*							
Bipolar kidney diameter (cm)	9.13	3.22	2.84	0.005	2.83	15.44	0.003
Parenchymal thickness (mm)	0.65	0.16	4.21	< 0.001	0.35	0.96	0.006
Gender (Male)	− 3.77	1.27	− 2.97	0.003	− 6.26	− 1.28	0.003
Proteinuria (g/day)	− 1.12	0.15	− 7.33	< 0.001	− 1.42	− 0.82	0.019
Clinical Diabetes (Yes)	− 7.47	2.22	− 3.37	0.001	− 11.82	− 3.13	0.004
Histopathological diagnosis				< 0.001			0.023
Histopathological diagnosis * bipolar kidney diameter interaction				0.004			0.017
Renal Pathology score (Ref. score 4)				0.006			0.005
RPS *n* = 1	28.80	12.29	2.34	0.019	4.70	52.89	0.002
RPS *n* = 2	19.37	7.30	2.66	0.008	5.07	33.68	0.003
RPS *n* = 3	7.96	7.07	1.13	0.261	− 5.91	21.82	0.000

Model adjusted R square of 0.219

In the Panel A, the adjusted R square value of the model increased consistently up to 0.216, with the major contribution made by the histopathological diagnosis (Eta square of 0.022) and with a significant interaction of histopathological diagnosis by longitudinal kidney diameter (P value = 0.006, Eta square of 0.017). In the Panel B, the addition of renal pathology score increased little the adjusted R square value (from 0.216 to 0.219) and, as expected, a high value of renal pathology score is associated with a reduction of eGFR

**Fig. 3:**
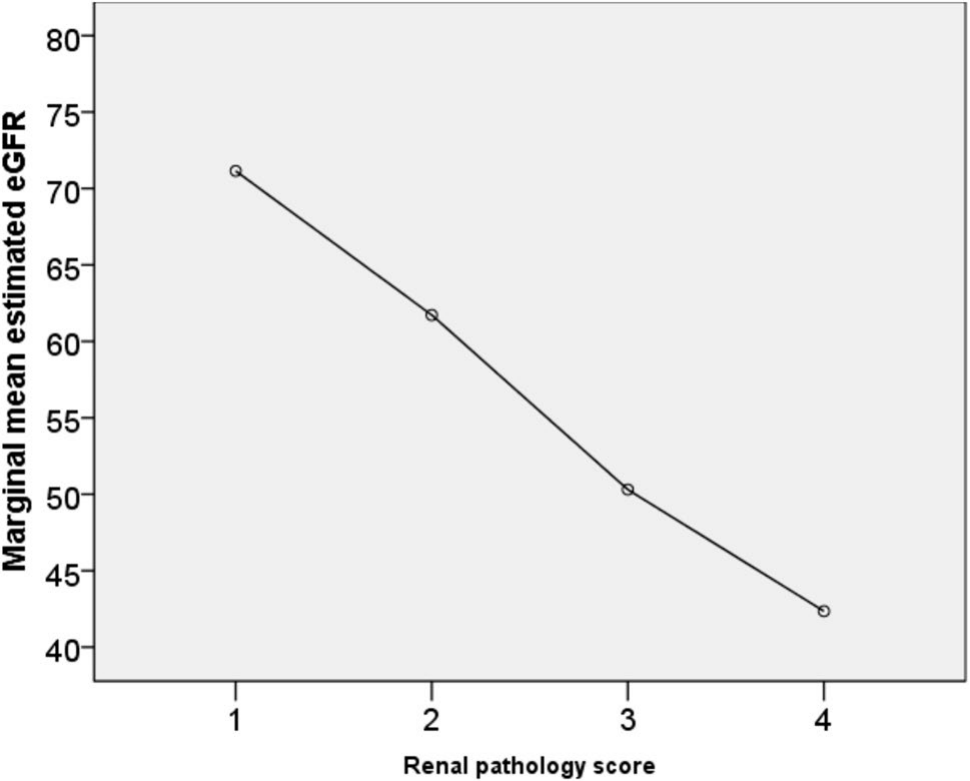
Inverse relationship between renal pathology score and marginal mean eGFR, after taking into the account the covariates of the multivariate analysis of the full model reported in the Panel B of Table 4. Renal Pathology score, according to reference [20], indicates the progressive severity of diabetic nephropathy from 1 to 4

Finally, the global model can be only slightly ameliorated, from 0.216 to 0.219, with the addition of renal pathology score, according to the pathologic classification of diabetic nephropathy [20] (Table 4, Panel B): as expected, a high value of renal pathology score was associated with a decrease of eGFR (Fig. 3).

## Discussion

The main findings of this study are (i) that is confirmed a direct relationship between eGFR and kidney mass, estimated with the kidney bipolar length and the parenchymal thickness, (ii) that diabetes status and proteinuria levels are associated inversely with the eGFR values, and finally (iii) that the association of kidney length with the level of eGFR is dependent on the nature of kidney disease.

Regarding the first finding, renal length and parenchymal thickness are clinically relevant parameters, often used for making clinical decisions [22]. In our study, both renal length and parenchymal thickness were associated directly with eGFR, with a major contribution of renal length (Eta square 0.037) compared with the parenchymal thickness (Eta square 0.006). As renal function loss occurs during the course of CKD, the measurement of kidney bipolar length using ultrasound can become a very useful tool and should be performed and reported in any clinical ultrasound kidney evaluation. Several studies have demonstrated the relationship between kidney size and eGFR in kidney donors [23], in renal transplant patients [24] and in older patients [25]. Since it is well-known that progressive loss of nephrons is associated with a reduction of kidney mass [26], correlations were performed between renal function in the elderlies and renal US parameters [27]. Apart from some specific kidney diseases such as polycystic kidney [28], longitudinal renal diameter is considered a pivotal marker of CKD, since it progressively declines together with GFR, thus with a direct relationship. Accordingly, polycystic kidney disease was absent in our sample, and also patients with acute kidney injury or acute kidney injury on chronic kidney disease, other confounding factors on the kidney diameter/eGFR relationship, were excluded from our analysis. Moreover, in most of the patients with acute kidney injury, renal US imaging shows normal or larger renal diameters [29]. For this reason, in our study, also patients with acute kidney injury were excluded.

In course of CKD, it is well known that there is a progressive loss of renal mass and a reduction of kidney length associated with a decline of GFR [4]. With age, this evolution pattern happens also in healthy subjects, in a less impressive manner, and can manifest differently in men and women [30]. Anyways, the relationship eGFR/kidney diameter length remained very weak in our study (adjusted R square value of 0.064) and this can be due to at least two limiting factors: the diameter length was measured only on one kidney, the biopsied one, and the lack of longitudinal observations. In fact, no information was collected on the contralateral kidney diameter. Regarding the latter limiting factor, the cross-sectional design of the study did not permit to take into account the progressive aging kidney atrophy, and the related kidney compensatory hypertrophy common in the CKD course [26, 31]. Kidney atrophy and subsequent opposite compensatory hypertrophy act in opposite directions on kidney length, with the final result of a reduced correlation between the kidney length and the eGFR.

The second relevant finding of our study was related to other two parameters easily available before biopsy, that can be used to improve the estimation of kidney damage: the clinical diabetes status and highest levels of proteinuria, that were associated with a significant and a consistent reduction of eGFR value, indeed the adjusted model R square value increased from 0.064 to 0.100. If the reduction of eGFR in diabetic patients at a late stage is an expected finding, the interaction of diabetes with ultrasound kidney parameters (bipolar length and parenchymal thickness) was not statistically significant (P value 0.389 and 0.819, respectively), meaning that the slope between the ultrasound kidney parameters and eGFR is not different between patients with and without clinical diabetes. Thus, for each incremental centimeter in renal length, we can expect the same increase of eGFR in both patients with and without clinical diabetes. This is another interesting finding of this study. Also, the association of high proteinuria with low eGFR levels, controlling for histopathological diagnosis, is another novel and interesting one. Thus, proteinuria has many roles, not only in various types of glomerulonephritis [32] and in Kidney Disease Screening Programs [33], but also in CKD staging, influencing directly the value of eGFR.

The third main finding of our study derived from the histopathological diagnosis of the biopsied kidney. In our study the association between histopathological diagnosis and the eGFR was confirmed. Indeed, including this variable in the multivariate analysis, the adjusted R square value increased consistently from 0.100 to 0.216. Moreover, there is a significant interaction of histopathological diagnosis with longitudinal kidney diameter (P = 0.006) suggesting that the association of kidney length with the level of eGFR is dependent on the nature of kidney disease.

Finally, this study had some strong points that are worth to be underlined. The opportunity to investigate the association between ultrasound parameters and eGFR, controlling, in a multivariate context, for the histopathological diagnosis is a very powerful and adequate study design. The ultrasound parameters were determined before kidney biopsy, and thus not dependent by bleeding risks of kidney biopsy [18] and/or by subsequent therapies suggested by the histopathological diagnosis. The large sample size and the combined availability of ultrasound kidney parameters with the histopathologic kidney diagnosis, validated and controlled by qualified histopathologists, are unique in the scientific literature, although its results are partially confirmatory.

## Conclusions

Renal bipolar length and cortical thickness are related directly with eGFR. Magnitude of proteinuria and histopathological kidney diagnosis are associated with eGFR values. The relationship between kidney length and the level of eGFR is dependent on the nature of kidney disease.

## Data Availability

Data may be shared upon reasonable request to the corresponding author.

## Appendix

See below Appendix Table 5 here.

**Table 5:** Cities, affiliations and names of collaborative authors of the ITA-KID-BIOPSY Group

ACIREALE, Ospedale Santa Marta e Santa Venera:	Maurizio Garozzo, Giovanni Giorgio Battaglia
AGRIGENTO, U.O.C Nefrologia e Dialisi San Giovanni di Dio:	Antonio Granata, Giulio Distefano, Monica Insalaco, Rosario Maccarrone
ALBANO LAZIALE, Ospedale Regina Apostolorum:	Marco Leoni, Angelo Emanuele Catucci, Emanuela Cavallaro
ALESSANDRIA, AOU "SS Antonio e Biagio e Cesare Arrigo":	Marco Quaglia, Brigida Brezzi
ANCONA, Umberto I:	Carolina Finale, Valentina Nastasi, Andrea Ranghino, Domenica Taruscia
AOSTA, Ospedale Regionale Umberto Parini:	Massimo Manes, Andrea Molino
ASCOLI PICENO, Ospedale “C. e G. Mazzoni”:	Giuseppe Fioravanti, Cinzia Fiori, Rosaria Polci
ASTI, S.C. Nefrologia e Dialisi Osp. Cardinal Massaia:	Olga Randone, Nicola Giotta, Stefano Maffei
BARI, Policlinico—Ist. Nefrologia:	Loreto Gesualdo, Annamaria Di Palma, Michele Rossini, Paola Suavo-Bulzis, Cosma Cortese, Carmen Sivo
BOLOGNA, IRRCS Azienda Ospedaliero-Universitaria, Nephrology, Dialysis and Kidney Transplant Unit, Sant'Orsola Hospital:	Gaetano La Manna, Olga Baraldi, Claudia Bini, Gisella Vischini
BORGOMANERO, Osp. SS. Trinità:	Luisa Benozzi, Stefano Cusinato
BRINDISI, Ospedale A. Perrino:	Luigi Vernaglione, Brigida Di Renzo, Cosima Balestra, Palmira Schiavone, Alessio Montanaro, Concetta Giovine
CASTELFRANCO VENETO, Osp. S. Giacomo:	Abaterusso Cataldo, Roberta Lazzarin
CATANIA, Nefrologia Cannizzaro:	Antonio Granata, Giuseppe Seminara, Daniela Puliatti
CHIETI, Policlinico “SS. Annunziata”:	Mario Bonomini, Roberto Di Vito, Teresa Lombardi, Luigia Larlori
CIRIÈ, Ospedale Civile:	Carolina Maria Licata, Vincenza Calitri
COMO, S. Anna:	Marco D'Amico, Beniamina Gallelli
CUNEO, Osp. S. Croce:	Elisabetta Moggia
EBOLI, Ospedale Maria Santissima Addolorata:	Giuseppe Gigliotti, Francesca Bruno, Pierluigi D'Angiò, Michele Nigro, Vincenzo Ragone
FERRARA, S. Anna:	Yuri Battaglia, Alda Storari, Sergio Sartori, Paola Tombesi
FOGGIA, Nefrologia Universitaria:	Barbara Infante, Giulia Godeas, Giovanni Stallone
FORLÌ, Morgani Pierantoni:	Marco De Fabritiis, Giovanni Mosconi
LA SPEZIA, Sant'Andrea:	Davide Rolla, Valentina Corbani, Francesca Lauria, Laura Panaro, Francesca Cappadona
LECCO, Associazione Italiana Ricercare per Curare ODV ETS (AIRpC):	Simeone Andrulli, Giovanni De Vito, Salvatore David e Giovanni Valsecchi
MESSINA, Policlinico-Nefrologia:	Domenico Santoro, Guido Gembillo, Antonello Salvo
MILANO, ASST Fatebenefratelli-Sacco, Università di Milano:	Nicoletta Landriani, Maurizio Gallieni, Gianmarco Sabiu
MODENA, Policlinico:	Francesco Fontana, Riccardo Magistroni, Ronni Luca Spaggiari, Francesca Testa, Gianni Cappelli
NAPOLI, Università della Campania—Nefrologia:	Giovambattista Capasso, Rosa Maria Pollastro, Davide Viggiano
NOVARA, Azienda Ospedaliero-Universitaria Maggiore della Carità:	Doriana Chiarinotti, Maria Maddalena Conte
NOVARA, Nephrology and Transplant Unit, "Maggiore della Carità" University Hospital, Università del Piemonte Orientale:	Marco Quaglia
NUORO, Ospedale San Francesco	Giovanni Mattana, Marilena Sedda, Anna Orru, Antonio Pais, Annalisa Sini, Agostina Angela Leoni, Francesco Logias
PALERMO, Ospedale Cervello:	Angelo Ferrantelli, Maria Giovanna Vario
PALERMO, UO di Nefrologia Dialisi e Trapianto ARNAS Civico:	Gioacchino Li Cavoli, Luisa Bono, Antonio Amato
PARMA, UOC Nefrologia, Azienda Ospedaliero Universitaria di Parma:	Lucio Manenti, Isabella Pisani, Monia Incerti
PERUGIA, Ospedale Santa Maria della Misericordia:	Rachele Brugnano, Raffaela Sciri, Ilaria Carriero
PESARO, Ospedali Riuniti Marche Nord:	Marina Di Luca, Flavia Manenti, Marco Palladino, Xhensila Gabrocka, Francesca Pizzolante
PISA, S. Chiara-Nefrologia:	Antonio Pasquariello, Giovanna Pasquariello, Maurizio Innocenti, Matilde Masini
PISA, U.O. Nefrologia Dialisi e Trapianti, Osp. CISANELLO:	Domenico Giannese
RAVENNA, S. Maria delle Croci:	Fulvia Zanchelli, Andrea Buscaroli, Mattia Monti
REGGIO EMILIA, S. Maria Nuova:	Enrica Gintoli, Elisa Gnappi, Mariacristina Gregorini
RIMINI, Osp. Degli Infermi:	Paola De Giovanni, Angelo Rigotti
ROMA, Policl. Umberto I:	Rosario Cianci, Antonietta Gigante
S. GIOVANNI ROTONDO, Fondazione Casa Sollievo della Sofferenza IRCCS:	Filippo Aucella, Matteo Piemontese, Michele Antonio Prencipe, Angela Maria Pellegrino
TARANTO, SS. Annunziata:	Luigi Morrone, Arcangelo Di Maggio
TORINO, Osp. Mauriziano:	Cristina Marcuccio
TORINO, Ospedale Martini:	Roberto Boero, Giulio Cesano
TRENTO, Presidio Ospedaliero Santa Chiara:	Laura Sottini, Giuliano Brunori
VERBANIA, Osp. Castelli:	Maurizio Borzumati, Maria Carmela Vella, Stefania Gioira
VERCELLI, S. Andrea:	Giovanna Mele, Luigia Costantini, Patrizia Colombo
VITERBO, Belcolle:	Sandro Feriozzi, David Micarelli, Franca Luchetta, Franco Brescia, Rossella Iacono

## References

[1] Spatola L, Andrulli S (2016) Doppler ultrasound in kidney diseases: a key parameter in clinical long-term follow-up. J Ultrasound 19(4):243–250. 10.1007/s40477-016-0201-x 27965714 10.1007/s40477-016-0201-x PMC5126008

[2] Singla RK, Kadatz M, Rohling R, Nguan C (2022) Kidney ultrasound for nephrologists: a review. Kidney Med 4(6):100464. 10.1016/j.xkme.2022.100464 35572095 10.1016/j.xkme.2022.100464 PMC9098467

[3] Stevens LA, Coresh J, Greene T, Levey AS (2006) Assessing kidney function–measured and estimated glomerular filtration rate. N Engl J Med 354(23):2473–2483. 10.1056/NEJMra054415 16760447 10.1056/NEJMra054415

[4] Levey AS, Inker LA, Coresh J (2014) GFR estimation: from physiology to public health. Am J Kidney Dis 63(5):820–834. 10.1053/j.ajkd.2013.12.006 24485147 10.1053/j.ajkd.2013.12.006 PMC4001724

[5] Cockcroft DW, Gault MH (1976) Prediction of creatinine clearance from serum creatinine. Nephron 16(1):31–41. 10.1159/000180580 1244564 10.1159/000180580

[6] Levey AS, Stevens LA, Schmid CH, Zhang YL, Castro AF 3rd, Feldman HI et al (2009) A new equation to estimate glomerular filtration rate. Ann Intern Med 150(9):604–612. 10.7326/0003-4819-150-9-200905050- 19414839 10.7326/0003-4819-150-9-200905050-00006 PMC2763564

[7] Michels WM, Grootendorst DC, Verduijn M, Elliott EG, Dekker FW, Krediet RT (2010) Performance of the Cockcroft-Gault, MDRD, and new CKD-EPI formulas in relation to GFR, age, and body size. Clin J Am Soc Nephrol 5(6):1003–1009. 10.2215/CJN.06870909 20299365 10.2215/CJN.06870909 PMC2879308

[8] Levey AS, Bosch JP, Lewis JB, Greene T, Rogers N, Roth D (1999) A more accurate method to estimate glomerular filtration rate from serum creatinine: a new prediction equation. Modification of Diet in Renal Disease Study Group. Ann Intern Med. 130(6):461–70. 10.7326/0003-4819-130-6-199903160-00002 10075613 10.7326/0003-4819-130-6-199903160-00002

[9] Levey AS, Coresh J, Greene T, Stevens LA, Zhang YL, Hendriksen S et al (2006) Using standardized serum creatinine values in the modification of diet in renal disease study equation for estimating glomerular filtration rate. Ann Intern Med 145(4):247–254. 10.7326/0003-4819-145-4-200608150-00004 16908915 10.7326/0003-4819-145-4-200608150-00004

[10] Inker LA, Schmid CH, Tighiouart H, Eckfeldt JH, Feldman HI, Greene T et al (2012) Estimating glomerular filtration rate from serum creatinine and cystatin C. N Engl J Med 367(1):20–29. 10.1056/NEJMoa1114248 22762315 10.1056/NEJMoa1114248 PMC4398023

[11] Inker LA, Eneanya ND, Coresh J, Tighiouart H, Wang D, Sang Y et al (2021) New creatinine- and cystatin C-based equations to estimate GFR without race. N Engl J Med 385(19):1737–1749. 10.1056/NEJMoa2102953 34554658 10.1056/NEJMoa2102953 PMC8822996

[12] Matsushita K, Mahmoodi BK, Woodward M, Emberson JR, Jafar TH, Jee SH et al (2012) Comparison of risk prediction using the CKD-EPI equation and the MDRD study equation for estimated glomerular filtration rate. JAMA 307(18):1941–1951. 10.1001/jama.2012.3954 22570462 10.1001/jama.2012.3954 PMC3837430

[13] Diao JA, Wu GJ, Wang JK, Kohane IS, Taylor HA, Tighiouart H et al (2023) National projections for of clinical implications race-free creatinine-based GFR estimating equations. J Am Soc Nephrol 34(2):309–321. 10.1681/ASN.2022070818 36368777 10.1681/ASN.2022070818 PMC10103103

[14] Fu EL, Coresh J, Grams ME, Clase CM, Elinder CG, Paik J et al (2023) Removing race from the CKD-EPI equation and its impact on prognosis in a predominantly White European population. Nephrol Dial Transplant 38(1):119–128. 10.1093/ndt/gfac197 35689668 10.1093/ndt/gfac197 PMC9869854

[15] Hundemer GL, White CA, Norman PA, Knoll GA, Tangri N, Sood MM et al (2022) Performance of the 2021 race-free CKD-EPI creatinine- and cystatin C-based estimated GFR equations among kidney transplant recipients. Am J Kidney Dis 80(4):462-472.e1. 10.1053/j.ajkd.2022.03.014 35588905 10.1053/j.ajkd.2022.03.014

[16] Shafi T, Zhu X, Lirette ST, Rule AD, Mosley T, Butler KR et al (2022) Quantifying individual-level inaccuracy in glomerular filtration rate estimation: a cross-sectional study. Ann Intern Med 175(8):1073–1082. 10.7326/M22-0610 35785532 10.7326/M22-0610

[17] Delanaye P, Radermecker RP, Rorive M, Depas G, Krzesinski JM (2005) Indexing glomerular filtration rate for body surface area in obese patients is misleading: concept and example. Nephrol Dial Transplant 20(10):2024–2028. 10.1093/ndt/gfh983 16030047 10.1093/ndt/gfh983

[18] Andrulli S, Rossini M, Gigliotti G, La Manna G, Feriozzi S, Aucella F et al (2023) The risks associated with percutaneous native kidney biopsies: a prospective study. Nephrol Dial Transplant 38(3):655–663. 10.1093/ndt/gfac177 35587882 10.1093/ndt/gfac177 PMC9976765

[19] Mazzucco G, Bertani T, Fortunato M, Bernardi M, Leutner M, Boldorini R et al (2002) Different patterns of renal damage in type 2 diabetes mellitus: a multicentric study on 393 biopsies. Am J Kidney Dis 39(4):713–720. 10.1053/ajkd.2002.31988 11920336 10.1053/ajkd.2002.31988

[20] Tervaert TW, Mooyaart AL, Amann K, Cohen AH, Cook HT, Drachenberg CB et al (2010) Pathologic classification of diabetic nephropathy. J Am Soc Nephrol 21(4):556–563. 10.1681/ASN.2010010010 20167701 10.1681/ASN.2010010010

[21] Lucisano G, Comi N, Pelagi E, Cianfrone P, Fuiano L, Fuiano G (2015) Can renal sonography be a reliable diagnostic tool in the assessment of chronic kidney disease? J Ultrasound Med 34(2):299–306. 10.7863/ultra.34.2.299 25614403 10.7863/ultra.34.2.299

[22] Cheong B, Muthupillai R, Rubin MF, Flamm SD (2007) Normal values for renal length and volume as measured by magnetic resonance imaging. Clin J Am Soc Nephrol 2(1):38–45. 10.2215/CJN.00930306 17699385 10.2215/CJN.00930306

[23] Donadio C, Abdelkawy H, Grassi G (2010) Echographic renal dimensions can predict glomerular filtration rate of potential living kidney donors. Transplant Proc 42(4):1035–1039. 10.1016/j.transproceed.2010.03.039 20534217 10.1016/j.transproceed.2010.03.039

[24] Paleologo G, Abdelkawy H, Barsotti M, Basha A, Bernabini G, Bianchi A et al (2007) Kidney dimensions at sonography are correlated with glomerular filtration rate in renal transplant recipients and in kidney donors. Transplant Proc 39(6):1779–1781. 10.1016/j.transproceed.2007.05.003 17692610 10.1016/j.transproceed.2007.05.003

[25] Akpinar IN, Altun E, Avcu S, Tüney D, Ekinci G, Biren T (2003) Sonographic measurement of kidney size in geriatric patients. J Clin Ultrasound 31(6):315–318. 10.1002/jcu.10178 12811791 10.1002/jcu.10178

[26] Kariyanna SS, Light RP, Agarwal R (2010) A longitudinal study of kidney structure and function in adults. Nephrol Dial Transplant 25(4):1120–1126. 10.1093/ndt/gfp654 19948878 10.1093/ndt/gfp654

[27] Van Den Noortgate N, Velghe A, Petrovic M, Vandewiele C, Lameire N, Voet D et al (2003) The role of ultrasonography in the assessment of renal function in the elderly. J Nephrol 16(5):658–662 14733411

[28] Grantham JJ, Cook LT, Torres VE, Bost JE, Chapman AB, Harris PC et al (2008) Determinants of renal volume in autosomal-dominant polycystic kidney disease. Kidney Int 73(1):108–116. 10.1038/sj.ki.5002624 17960141 10.1038/sj.ki.5002624 PMC2790405

[29] O’Neill WC (2000) Sonographic evaluation of renal failure. Am J Kidney Dis 35(6):1021–1038. 10.1016/s0272-6386(00)70036-9 10845813 10.1016/s0272-6386(00)70036-9

[30] Piras D, Masala M, Delitala A, Urru SAM, Curreli N, Balaci L et al (2020) Kidney size in relation to ageing, gender, renal function, birthweight and chronic kidney disease risk factors in a general population. Nephrol Dial Transplant 35(4):640–647. 10.1093/ndt/gfy270 30169833 10.1093/ndt/gfy270 PMC7139213

[31] Lee D, Levin A, Roger SD, McMahon LP (2009) Longitudinal analysis of kidney structure and function in adults. Nephrol Dial Transplant 24(1):109–116. 10.1093/ndt/gfn477 18755849 10.1093/ndt/gfn477

[32] Pozzi C, Andrulli S, Del Vecchio L, Melis P, Fogazzi GB, Altieri P et al (2004) Corticosteroid effectiveness in IgA nephropathy: long-term results of a randomized, controlled trial. J Am Soc Nephrol 15(1):157–163. 10.1097/01.asn.0000103869.08096.4f 14694168 10.1097/01.asn.0000103869.08096.4f

[33] Imai E, Yamagata K, Iseki K, Iso H, Horio M, Mkino H et al (2007) Kidney disease screening program in Japan: history, outcome, and perspectives. Clin J Am Soc Nephrol 2(6):1360–1366. 10.2215/CJN.00980207 17942780 10.2215/CJN.00980207

